# Leucine‐rich repeat‐containing G protein‐coupled receptor 5 expression in lymph node metastases of colorectal cancer: Clinicopathological insights and prognostic implications

**DOI:** 10.1111/pin.13439

**Published:** 2024-05-24

**Authors:** Hiroshi Sawaguchi, Takeshi Uehara, Mai Iwaya, Shiho Asaka, Tomoyuki Nakajima, Masato Kamakura, Tadanobu Nagaya, Takahiro Yoshizawa, Hiroyoshi Ota, Takeji Umemura

**Affiliations:** ^1^ Department of Medicine, Division of Gastroenterology and Hepatology Shinshu University School of Medicine Matsumoto Japan; ^2^ Department of Laboratory Medicine Shinshu University School of Medicine Matsumoto Japan; ^3^ Department of Laboratory Medicine Nagano Children's Hospital Azumino Japan; ^4^ Division of Gastroenterological, Hepato‐Biliary‐Pancreatic, Transplantation and Pediatric Surgery, Department of Surgery Shinshu University School of Medicine Matsumoto Japan; ^5^ Department of Biomedical Laboratory Medicine Shinshu University School of Medicine Matsumoto Japan

**Keywords:** colorectal cancerl, eucine‐rich repeat‐containing G‐protein‐coupled receptor 5, lymph node metastasis, RNA in situ hybridization

## Abstract

Leucine‐rich repeat‐containing G protein‐coupled receptor 5 (*LGR5*), a significant cancer stem cell marker in colorectal cancer (CRC), lacks lymph node (LN) expression studies. In this study, we identified *LGR5* expression by RNAscope, a highly sensitive RNA in situ method, and analyzed its association with clinicopathological characteristics. Tissue microarrays were generated from primary tumors (PTs) and LN metastases in paraffin‐embedded blocks of 38 CRC surgical resection materials. *LGR5* expression by RNAscope was evaluated by dividing the expression levels into negative and positive expression. In all but two cases of LN metastasis, *LGR5*‐positive dots were detected in tumor cells, and there was a wide range of *LGR5*‐positive cells. More *LGR5*‐positive dots were identified in the gland‐forming region. Twenty‐three cases were classified into a high *LGR5*‐expression group, and 15 cases were classified into a low *LGR5*‐expression group. In the high LGR5‐expression group, the histological grade was lower than in the low *LGR5*‐expression group (*p* = 0.0159), while necrosis was significantly more prevalent (*p* = 0.0326), and the tumor, node, metastasis stage was significantly lower (*p* = 0.0302). There was no association between *LGR5* expression levels in LN metastases and LGR5 expression levels in PT tissue. *LGR5* expression in LN metastases may influence prognosis. Further analysis may lead to new therapeutic strategies.

**Abbreviations**
CRC: colorectal cancer
CSC: cancer stem cell
EMT: epithelial–mesenchymal transition
ISH: in situ hybridization
LGR5: leucine‐rich repeat‐containing G protein‐coupled receptor 5
OS: overall survival
TMA: tissue microarray

## INTRODUCTION

Colorectal cancer (CRC) is a malignant tumor of the gastrointestinal tract that ranks among the top causes of cancer‐related death worldwide. Despite surgical and anticancer therapies, the clinical course of the disease still has a poor prognosis and ranks third in both new cases and deaths in US statistics.^1^ Globally, the CRC burden is projected to increase by approximately 60% by 2030, and there are concerns about increasing morbidity and mortality, especially in developing countries.^2^ Therefore, new information on CRC prognostication and metastasis may be important in determining future treatment plans and directions.

Leucine‐rich repeat‐containing G protein‐coupled receptor 5 (*LGR5*) is a seven‐transmembrane receptor and a target gene for Wnt/β‐catenin signaling. *LGR5* has been identified in various studies as a marker for biological stem cells in the small and large intestines and in hair follicles.^3^, ^4^ The Wnt/β‐catenin signaling pathway is involved in various cellular functions, including cell proliferation and migration, and is often dysregulated in cancer.^5^ LGR5 is closely related to the regulation of Wnt/β‐catenin signaling and also plays an important role in the regulation of cancer stem cell (CSC) function.^6^, ^7^, ^8^ Several papers have also reported that LGR5 is overexpressed in primary tumors (PTs) in CRC.^9^, ^10^ Reports on the prognostic value of CRC and LGR5 expression have shown that the higher the expression, the worse the prognosis, and a number of meta‐analyses have concluded that LGR5 expression is a poor prognostic factor for CRC.^11^, ^12^, ^13^, ^14^ These studies have primarily used immunohistochemical analysis to assess LGR5 expression; however, useful antibodies against LGR5‐positive cells are fairly limited.^15^, ^16^ Nonetheless, RNA in situ hybridization (ISH) has been recognized as a good method for detecting the presence of *LGR5* expression^17^. There have been reports of similar studies using ISH methods with the exact opposite results for *LGR5* expression and prognosis, so there remains much room for debate regarding *LGR5* expression and the prognosis of CRC.^18^

Although there have been several studies on stem cell marker expression and prognosis in lymph node (LN) metastases of CRC, reports on *LGR5* analysis are limited. Therefore, we used RNAscope, a sensitive RNA INH method, to identify *LGR5* expression in LN metastases and to analyze clinicopathological characteristics. Investigation of the relationship between *LGR5* expression and prognosis in LN metastasis may allow for targeted therapy in LN metastases, as well as prognosis prediction and potential targeted therapy in CRC.

## MATERIALS AND METHODS

### Patients

Two hundred and fifty cases of primary CRC were resected at Shinshu University from 2014 to 2021, including 76 cases with LN metastasis. Patients were followed for at least 2 years. Because we focused on *LGR5* expression by tumor differentiation level, well differentiated, moderately differentiated, and poorly differentiated adenocarcinomas were selected. In accordance with previous reports,^19^ well and moderately differentiated adenocarcinomas were classified as low grade, and poorly differentiated as high grade. Therefore, 20 cases containing other histological types were excluded. Among these patients, 68 cases were excluded for the following reasons: 67 cases were negative for the positive control (housekeeping gene) in the PT or LN metastases or both and one case had no tumor tissue at the primary site within a tissue microarray (TMA). Ultimately, 162 cases of CRC including 38 cases of LN metastases were enrolled. Data on patient age and sex, pathological differentiation, and tumor, node, metastasis (TNM) classification were obtained by review of medical records. Clinical stage and tumor differentiation were determined using the 8th UICC classification^20^ and the 5th edition of the World Health Organization classification.^21^ Histological characteristics of all specimens were confirmed by two pathologists (Takeshi Uehara and Mai Iwaya). Overall survival (OS) was defined as the interval between the date of surgical resection and the date of death or last follow‐up. This study was conducted in accordance with the current ethical guidelines of the Declaration of Helsinki and the requirements of the Shinshu University School of Medicine Clinical Trial Review Committee (approval no. 5836).

### Histopathology and TMA construction

All specimens were fixed in 10% or 20% formaldehyde and embedded in paraffin. Blocks for the construction of a TMA were selected from formalin‐fixed paraffin‐embedded tissue that contained sufficient tumor tissue and LN metastases. Tissue cores were punched out from each block using thin‐walled 3‐mm stainless steel needles (Azumaya Medical Instruments Inc.), and cores were arrayed in a recipient paraffin block. Serial sections of 4‐µm thickness were cut from these blocks and stained with hematoxylin‐eosin.

### *LGR5* RNA ISH

Detection of *LGR5* mRNA was performed using an RNAscope kit (Advanced Cell Diagnostics) according to the manufacturer's instructions using unstained sample tissue slides. Briefly, tissue sections were pretreated with heating and protease application prior to hybridization with an *LGR5*‐specific probe. The detailed procedure is described in a previous report.^22^ Brown punctate dots present in the nucleus and/or cytoplasm indicated positive staining. *LGR5* expression was quantified according to the five‐grade scoring system recommended by the manufacturer (no staining, 0; 1–3 dots/cell, 1+; 4–9 dots/cell, 2+; 10–15 dots/cell, 3+; and >15 dots/cell, 4+) under a 20× objective lens (Olympus BX53). Furthermore, *LGR5* mRNA expression was categorized as low *LGR5*‐expression (grade 0, 1+, and 2+) or high *LGR5*‐expression (3+ and 4+). We analyzed the relationship between *LGR5* expression and clinicopathological data and prognosis in LN metastasis.

### Statistical analysis

For clinicopathological characteristics, categorical variables were expressed as a number. Pearson's chi‐squared tests were adopted to test for differences between subgroups of patients. The OS rates of CRC patients were calculated using the Kaplan–Meier method, and differences in those rates were compared by the log‐rank test. Univariate analyses for prognostic factors were performed using a Cox proportional hazard regression model. A *p*‐value of <0.05 was considered significant. All statistical analyses were performed using JMP Statistics software version 13 (JMP).

## RESULTS

### *LGR5* expression in LN metastasis

**Figure 1:**
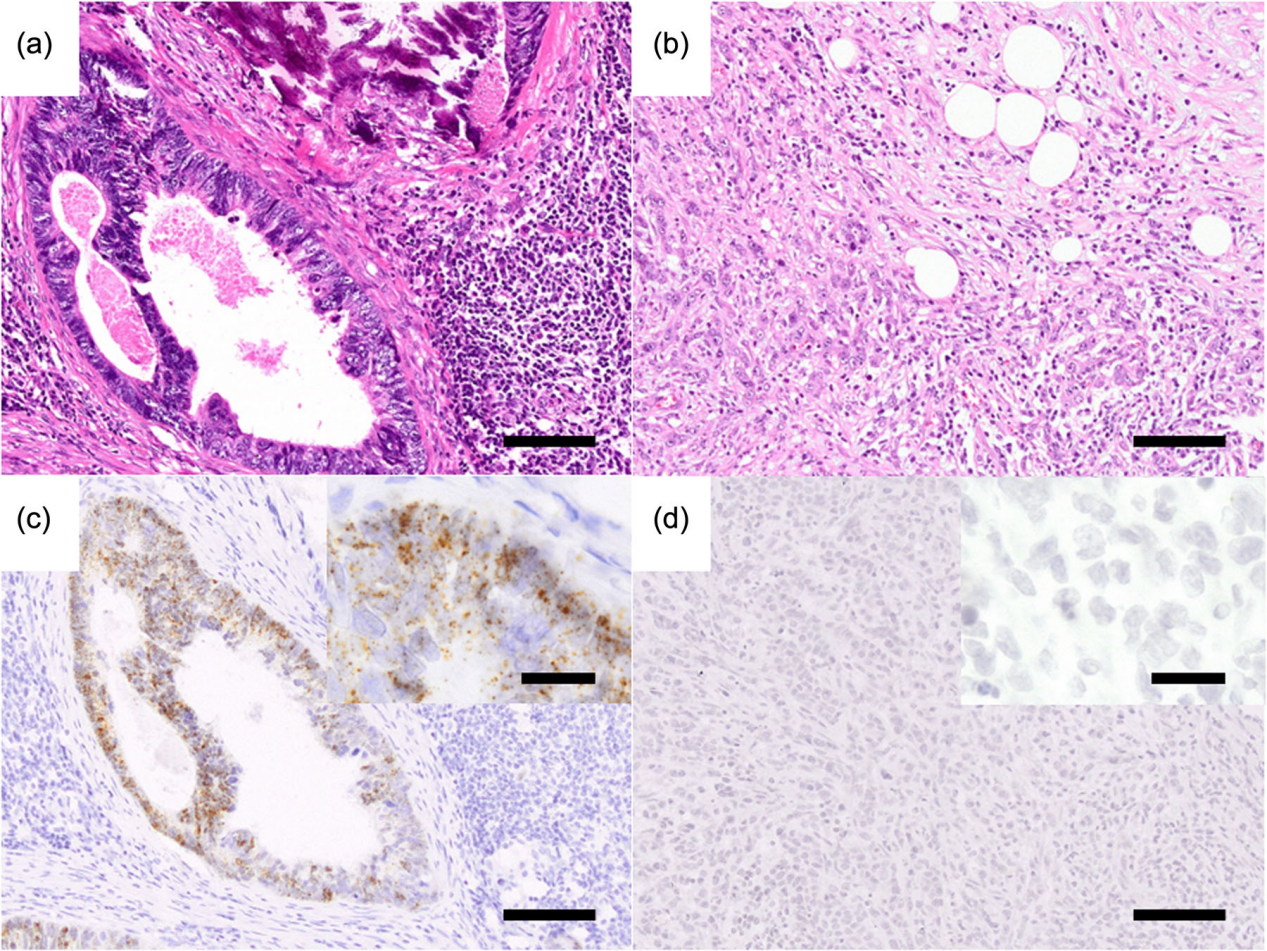
Leucine‐rich repeat‐containing G protein‐coupled receptor 5 (LGR5)‐expression in lymph node (LN) metastasis. Representative features in lymph nodes with low‐grade adenocarcinoma (a) and high‐grade adenocarcinoma (b). In lymph nodes with low‐grade adenocarcinoma, high *LGR5*‐expression was observed (c). However, in LNs with high‐grade adenocarcinoma, low *LGR5*‐expression was observed (d). [(a, b), hematoxylin‐eosin; (c, d), *LGR5* RNAscope]. Bar indicates 100 μm (magnified panel = 20 μm).

In all but two cases of LN metastasis, *LGR5*‐positive dots were detected in tumor cells, and there was a wide range of *LGR5*‐positive cells. More *LGR5*‐positive dots were identified in the gland‐forming region. Twenty‐three cases were classified into a high *LGR5*‐expression group (Figure 1a,c) and 15 cases were classified into a low *LGR5*‐expression group (Figure 1b,d).

### *LGR5* expression and clinicopathological characteristics in LN metastasis

The clinicopathological characteristics of the patients with LN metastasis are described in Table 1. In the high *LGR5*‐expression group, histological grade was lower than that of the low *LGR5*‐ expression group (*p* = 0.0159). However, no significant differences were identified between *LGR5* expression and clinicopathological features in PT (Table 2).

**Table 1:** *LGR5* expression in LN and clinicopathological characteristics of patients with colorectal cancer and lymph node metastasis.

		*LGR5‐*expression in LN		
Factors	*n*	High (*n* = 23)	Low (*n* = 15)	*p*‐Value
Age				0.1000
>70 years	17	13	4	
≤70 years	21	10	11	
Sex				1.0000
Male	23	14	9	
Female	15	9	6	
Vascular invasion in PT				0.1143
Present	30	16	14	
Absent	8	7	1	
Histological grade in LN				**0.0159**
Low	29	21	8	
High	9	2	7	
Necrosis in LN				**0.0326**
Present	26	19	7	
Absent	12	4	8	
*LGR5‐*expression in PT				0.7356
Low	23	13	10	
High	15	10	5	
*N* stage				0.3235
1a–1b	16	8	8	
2a–2b	22	15	7	
TNM stage				**0.0302**
III	10	3	7	
IV	28	20	8	

*Note*: Bold *p*‐Values are statistically significant.

Abbreviations: LGR5, leucine‐rich repeat‐containing G protein‐coupled receptor 5; LN, lymph node; PT, primary tumor; TNM, tumor, node, metastasis.

**Table 2:** *LGR5* expression in LN and clinicopathological characteristics in patients with colorectal cancer and LN metastasis.

		*LGR5‐*expression in PT		
Factors	*n*	High (*n* = 15)	Low (*n* = 23)	*p*‐Value
Age				0.1853
>70 years	17	9	8	
≤70 years	21	6	15	
Sex				0.7356
Male	23	10	13	
Female	15	5	10	
Vascular invasion in PT				0.6869
Present	30	11	19	
Absent	8	4	4	
Histological grade in PT				1.0000
Low	34	14	20	
High	4	1	3	
Histological grade in LN				1.0000
Low	29	11	18	
High	9	4	5	
Necrosis in LN				0.1569
Present	26	8	18	
Absent	12	7	5	
*N* stage				1.0000
1a–1b	16	6	10	
2a–2b	22	9	13	
TNM stage				0.1499
III	10	6	4	
IV	28	9	19	

Abbreviations: LGR5, leucine‐rich repeat‐containing G protein‐coupled receptor 5; LN, lymph node; PT, primary tumor; *N* stage, regional lymph node involvement stage; TNM, tumor, node, metastasis.

In the high *LGR5*‐expression group, necrosis was significantly more common than that in the low *LGR5*‐expression group (*p* = 0.0326). In the high *LGR5*‐expression group, TNM stage was significantly lower than that of the low *LGR5*‐expression group (*p* = 0.0302). There was no significant difference between the high *LGR*5‐expression group and the low *LGR5*‐expression group in terms of age, sex, or vascular invasion. There was no association between *LGR5* expression levels in LN metastases and in PT tissue.

Among patients with high *LGR5* expression in PT, those with low *LGR5* expression in LN had less necrosis than those with high *LGR5* expression (*p* = 0.007). No significant differences were found in age, sex, vascular invasion, histological grade, N stage, or TNM stage (*p* = 0.3287, *p* = 1, *p* = 1, *p* = 0.6004, *p* = 0.0889, and *p* = 0.0889, respectively). However, among the patients with low *LGR5* expression in PT, those with high *LGR5* expression in LN was not significantly characterized by age, sex, vascular invasion, histological grade, necrosis, *N* stage, or TNM stage (*p* = 0.3788, *p* = 1, *p* = 0.1045, *p* = 1, *p* = 0.6175, *p* = 1, and *p* = 0.2806, respectively) compared with those with low *LGR5* expression in LN.

### Prognostic value of *LGR5* in LN metastasis

We assessed the prognostic value of *LGR5* expression in lymph node metastasis by Kaplan–Meier analysis and the log‐rank test. The median OS rate for the study patients was 32 (range, 19.75–51.5) months. There was no significant difference in OS between the cases in the high *LGR5*‐expression group (median OS, 30 [range: 16–51] months) and the low *LGR5*‐expression group (median OS, 32 [range: 20–53] months) (log rank test, *p* = 0.7230).

*LGR5* expression did not remain a predictor of prognosis in the univariate analysis (*p* = 0.7168) (Table 3).

**Table 3:** Univariate analysis of overall survival factors in patients with colorectal cancer and lymph node metastasis.

	Univariate analysis
Factors	*p*‐Value
Age: >70 years versus ≤70 years	0.6810
Sex: male versus female	0.3848
Vascular invasion: absent versus present	0.9294
Histological grade in PT: low versus high	0.2072
Necrosis: absent versus present	0.9014
*N* stage: 1a–1b versus 2a–2b	0.2369
TNM stage: III versus IV	**0.0228**
*LGR5‐*expression in LN: low versus high	0.7168
*LGR5‐*expression in PT: low versus high	0.1099

*Note*: Bold *p*‐Value is statistically significant.

Abbreviations: LGR5, leucine‐rich repeat‐containing G protein‐coupled receptor 5; LN, lymph node; PT, primary tumor; *N* stage, regional lymph node involvement stage; TNM, tumor, node, metastasis.

## DISCUSSION

*LGR5* expression in LN metastasis was higher in cases with low‐grade histology and lower stage. These are new findings and may indicate that evaluation of *LGR5* expression in LN metastasis is a good prognostic tool. Genomic instability is a hallmark of cancer, and certain populations of tumor cells have a propensity to metastasize. As a result, metastasis may occur, and tumor cells at metastatic sites may acquire additional new genetic mutations. It has been suggested that critical features that could further progress cancer may be acquired as the disease progresses.^23^ Significant genetic differences may exist between PTs and metastases. Therefore, it may be more optimal to predict a patient's treatment response and prognosis based on the biological characteristics of the metastases.^24^ Evaluation of *LGR5* expression in LN metastasis may predict more distant metastases.

*LGR5* exhibits plasticity, and when CRC metastasizes, *LGR5* is negatively transformed, and *LGR5* expression is re‐expressed at the metastasis site.^25^ These reports may reinforce our study. In other words, they may explain why clinicopathological features associated with *LGR5* expression in LN metastasis were not identified in PTs. The high *LGR5* expression in carcinoma with necrosis may be involved in tumor tissue regeneration. It has been reported that LGR5 expression is elevated in the vicinity of necrosis.^26^

Although there are conflicting views regarding the prognosis with *LGR5* expression, there are several reports of favorable prognosis. LGR5 expression suppresses metastasis via transforming growth factor‐β.^24^ In ovarian cancer, *LGR5* expression is a favorable prognostic factor.^27^ *LGR5* expression also suppresses epithelial–mesenchymal transition (EMT) by acting on Wnt/β‐catenin.^18^ Because EMT is strongly implicated in metastasis and prognosis, regulation of *LGR5* expression may inhibit metastasis. *LGR5* is an intestinal tissue‐specific stem cell signature^28^; hence, it may be more abundantly expressed in adenocarcinomas that show intestinal differentiation such as gland formation.^29^ Thus, *LGR5* may have different biological characteristics than more primitive CSC markers.

*LGR5* is also associated with methylation abnormalities in cancer. It has been reported that when Lgr5 expression is reduced by cytosine‐phosphate‐guanosine island methylation, the expression of Snail, lug, Zeb1, and *N*‐cadherin is high and the expression of epithelial‐related genes such as *Cdh1* is reduced, leading to induction of EMT and metastatic processes.^30^ Furthermore, *LGR5* has a promoter methylated in CRC.^28^

*LGR5* expression is associated with histology in LN metastasis and may also be influenced by the surrounding environment. In the association between LGR5 and EMT, LGR5 enhances E‐cadherin expression via IQGAP1,^31^ even though *LGR5* does not act as an RSPO ligand. In other words, *LGR5* expression may be less likely to cause EMT. Therefore, even in the absence of poorly differentiated cells, reduced *LGR5* expression may result in a predisposition to EMT and poor prognosis. However, *LGR5* expression may be affected by the tumor microenvironment. In fact, the tumor microenvironment has been shown to influence CSC maintenance.^32^ Therefore, it is possible that the tumor environment and *LGR5* expression interact with each other, which may differ between PTs and metastases.

*LGR5* expression and the surrounding environment in metastases such as LN metastases have not been studied extensively. The relationship between *LGR5* expression and cytokine expression in the microenvironment should be analyzed by visualization using spatial transcriptomics and by co‐culture. In addition, the RNAscope method was used for relatively recently diagnosed cases, and hence the prognostic follow‐up was not sufficient. Long‐term prognostic impact should also be analyzed. The correlation between clinical stage and *LGR5* expression in LN metastasis may allow us to speculate on the prognostic impact of *LGR5* expression. Tumor differentiation in LN metastasis has been reported to influence prognosis,^33^ and its correlation with *LGR5* expression may have prognostic significance.

We have identified clinicopathological features of *LGR5* expression in LN metastasis. Further studies on the association between *LGR5* expression in LN metastasis and prognosis are warranted. Further analyses may lead to new therapeutic strategies.

## AUTHOR CONTRIBUTIONS

Hiroshi Sawaguchi participated in the design of the study, performed the pathological analysis, and drafted the manuscript. Takeshi Uehara and Mai Iwaya helped perform the pathological analysis. Takeshi Uehara performed the statistical analysis. Hiroshi Sawaguchi and Tomoyuki Nakajima performed the TMA construction and RNAscope. Masato Kamakura, Tadanobu Nagaya, and Takahiro Yoshizawa examined the clinical data of cases. Takeshi Uehara, Mai Iwaya, Shiho Asaka, Hiroyoshi Ota, and Takeji Umemura critically revised the draft. All authors have read and approved the manuscript.

## CONFLICT OF INTEREST STATEMENT

The authors declare no conflict of interest.

## ETHICS STATEMENT

The Ethics Committee of Shinshu University School of Medicine approved this study (approval code: 5836). The requirement of informed consent was waived by the ethics committee of Shinshu University School of Medicine, and an opt‐out method was used because of the retrospective design of the study. The investigation was conducted in compliance with the Declaration of Helsinki.

## Data Availability

All data generated and analyzed during the current study are available from the corresponding author upon reasonable request.
